# First successful protocol for desensitization to eptinezumab

**DOI:** 10.1111/head.70000

**Published:** 2025-12-19

**Authors:** Benoit Gerard, Hubert Praudel, Michel Lanteri‐Minet, Elise Van Obberghen, Fanny Rocher‐Moreau, Johanna Rousset, Ulysse Jacquier, Sylvie Leroy, Margot Delin

**Affiliations:** ^1^ Département de pneumologie et d'oncologie thoracique, Centre Hospitalier Universitaire de Nice, IHU RespirERA Université Côte d'Azur Nice France; ^2^ Pain Department, Centre Hospitalier Universitaire de Nice, FHU Inovpain, URC2A Université Côte d'Azur Nice France; ^3^ INSERM U1107 Migraine and Trigeminal Pain Auvergne University Clermont‐Ferrand France; ^4^ Centre régional de pharmacovigilance, Centre Hospitalier Universitaire de Nice Université Côte d'Azur Nice France

**Keywords:** allergy, desensitization, eptinezumab, skin test

## Abstract

**Background:**

Eptinezumab is an anti‐calcitonin gene‐related peptide monoclonal antibody used for migraine prevention. During clinical trials, hypersensitivity to eptinezumab was described without a clear underlying mechanism. To determine if the reaction was immunoglobulin E–mediated, the team of Nice University Hospital (Nice, France) performed the eptinzeumab skin tests. A 10‐step desensitization protocol has been proposed to manage these reactions.

**Findings:**

Two patients presenting with immediate hypersensitivity reactions underwent the eptinezumab skin test. Neither skin test was positive, suggesting a non‐immunoglobulin E–mediated mechanism. The patients then completed the desensitization protocol successfully.

**Conclusion:**

This hospital‐based 10‐step desensitization protocol appears safe and effective.

## INTRODUCTION

Eptinezumab, an intravenous anti‐calcitonin gene‐related peptide (CGRP) monoclonal antibody that is administered quarterly for migraine prevention, demonstrated a favorable safety profile in pivotal trials.^1^ However, in these trials, hypersensitivity reactions were observed in 15 (0.7%) patients among a pooled population of 2076 patients with migraine treated with eptinezumab.^1^ Reported hypersensitivity reactions included urticaria, flushing, rash, pruritus, and rarely anaphylaxis. The incidence of hypersensitivity was greater in a subsequent trial involving patients with migraine for whom standard oral prophylaxis failed. Hypersensitivity was observed in six (2%) and 10 (3%) patients among 299 patients with migraine treated with eptinezumab 100 mg, and among 294 patients with migraine treated with eptinezumab 300 mg, respectively.^2^ These hypersensitivity reactions were reported without mechanistic investigation. Drug hypersensitivity reactions were divided in four major pathophysiologic categories based on immunologic mechanism—type I: immediate (immunoglobulin [Ig]E‐mediated); type II: cytotoxic (IgG‐mediated); type III: immune complex disease (IgG‐ or IgM‐mediated); and type IV: delayed (cell‐mediated).^3^ Only type I and type IV reactions could be tested through skin testing.^3^ To date, no solution has been given to patients who have experienced such hypersensitivity reactions, other than treatment discontinuation, which is highly detrimental to patients with migraine who did not respond to standard treatments and positively responded to this biotherapy.

To date, no desensitization protocol to eptinezumab has been published. Analysis of pharmacovigilance data has been performed, including a specific request to the national French pharmacovigilance database and to the World Health Organization (WHO) international pharmacovigilance database on the June 4, 2025 (VigiBase), but there was no identified case describing induction of a tolerance protocol.

We report two cases of immediate hypersensitivity reactions in women with chronic migraine who successfully underwent hospital‐based desensitization, after allergy investigations, demonstrating the clinical feasibility of this approach.

Both patients gave their written informed consent to publish their case report.

## CASES SERIES

### Case 1

A 36‐year‐old woman nurse with no allergic past medical history presented with disabling chronic migraine, refractory to multiple preventive therapies (propranolol, amitriptyline, topiramate, and flunarizine).

In 2023, the patient began eptinezumab prophylaxis (100 mg IV over 30 min infusion every 3 months). The first infusion was well tolerated. She had an excellent response to eptinezumab because it reduced her frequency of migraine days by 75%. After the second dose, she developed a pruritic erythematous rash on both forearms 2.5 h postinfusion; of note, no NSAID was taken concomitantly. The rash resolved with cetirizine within 48 h without any mucosal or systemic involvement. For the third dose, premedication with cetirizine 10 mg was administered but a more extensive rash appeared 45 min postinfusion, again resolving with antihistaminic treatment. In both incidents, tryptase level was not measured. Tryptase dosage is recommended in drug hypersensitivity reaction to determinate an IgE‐mediated mechanism. In case of type I reaction, tryptase showed a 120% increase from baseline.

Intradermal skin tests (8 months after initial dose) were negative for eptinezumab at concentrations ranging from 10^−3^ to 10^−1^ with a pure 100 mg/mL solution. Desensitization was performed using a standardized protocol (Table 1) as per European safety recommendations such as emergency trolley, staff trained in resuscitation with intensive care facilities nearby, safety infusion, installation in a dedicated area, monitoring of vital signs before each new level, maximal increase between steps is doubling dose, and time between each step is around 20 to 30 min.^4^ Though delayed localized erythema appeared within 72 h with moderate pruritus, it resolved within 48 h using cetirizine. Subsequent desensitizations (3‐month intervals) were progressively better tolerated, with no further reactions since the third desensitization.

**TABLE 1:** Eptinezumab desensitization protocol for both cases.

Premedication 30 min before: dexchlorpheniramine 5 mg, methylprednisolone 60 mg						
Step	Concentration (mg/mL)	Duration (min)	Infusion rate (mL/h)	Volume (mL)	Dose administered (mg)	Cumulative dose (mg)
1	0.10	20 (15)	3 (4)	1	0.1	0.1
2	0.10	20 (15)	6 (8)	2	0.2	0.3
3	0.10	20 (15)	12 (16)	4	0.4	0.7
4	0.10	20 (15)	24 (32)	8	0.8	1.5
5	1.00	20 (15)	5 (6)	1.5	1.5	3
6	1.00	20 (15)	9 (12)	3	3	6
7	1.00	20 (15)	18 (24)	6	6	12
8	1.00	20 (15)	36 (48)	12	12	24
9	10.0	60 (40)	3 (4)	2.5	25	49
10	10.0	60 (40)	5 (8)	5	50	99
Total		4 h 40 (3 h 30)				99

*Note*: After three well‐tolerated desensitization administrations, protocol was accelerated by reducing administration time and increasing dose of several steps (modifications are highlighted in gray).

### Case 2

A 26‐year‐old woman student presented with moderate asthma, anxiety, and depression, as well as renal lithiasis. Her daily treatment consisted of montelukast, formoterol/budesonide, and duloxetine. She had chronic migraine refractory to multiple preventive therapies (propranolol, amitriptyline, topiramate, pizotifen, flunarizine, onabotulinumtoxinA injections, and occipital nerve block).

The patient's first eptinezumab infusion triggered generalized urticaria with immediate bronchospasm requiring oxygen, dexchlorpheniramine 4 mg, and salbutamol; no NSAIDs were taken. Because eptinezumab was effective for migraine, a second dose (3 months after the initial dose) under premedication with cetirizine (10 mg) and intravenous methylprednisolone (1 mg/kg) was tried. Generalized urticaria appeared 30 min postinfusion, without systemic involvement, and resolved within 48 h with cetirizine 10 mg/day. In both incidents, tryptase level was not measured. Intradermal eptinezumab skin tests were negative (same dilutions as in case 1). Desensitization was performed using the same protocol (Table 1), with no adverse events. No adverse event occurred for the next three desensitization courses, allowing the protocol to be accelerated after the third desensitization (Table 1; see gray highlighting).

## DISCUSSION

These two cases presented with immediate hypersensitivity reactions to IV eptinezumab without evidence of IgE‐mediated hypersensitivity given the negative skin tests. As a reminder, immediate drug hypersensitivity reactions are likely type I reactions, and thus IgE‐mediated. Immediate skin tests are used when a medical history is involved to explore IgE‐mediated diseases. There are two types of skin testing used in clinical practice: percutaneous testing (prick or puncture) and intracutaneous testing (intradermal). A positive skin testing with an immediate hypersensitivity history confirmed the diagnosis of type I allergy (IgE‐mediated)/anaphylaxis. It is clinically important to know if the reaction is anaphylaxis because it is life‐threatening or dose‐independent or because the initial reaction gets worse every time the patient is exposed again.^3^ Trying a desensitization protocol is justified when it proves beneficial to patients and after failure of premedication reintroduction. In our first case, the patient had a minor reaction after the first desensitization protocol, and the situation improved as the protocol was pursued. In the second case, the patient experienced tolerance during the cure, which helped speed up the protocol. Successive administrations potentially promote tolerance. As a humanized monoclonal antibody, eptinezumab has a modified fragment crystallizable (Fc) region that minimizes complement activation and antibody‐dependent cytotoxicity. Additionally, its soluble target (CGRP) also reduces immune complexes deposition in tissues. However, complement activation via anaphylatoxins (C3a/C5a) remains possible in the presence of soluble immune complexes.^5^ Alternative non–IgE‐mediated mechanisms, such as reactions to the excipient polysorbate 80 (a known trigger of pseudoallergic reactions with other monoclonal antibodies), may also contribute to the observed symptoms.^6^ Exposure to high doses of polysorbate 80 causes activation of nonspecific basophils and therefore hypersensitivity reactions. In this case, the patient could have had similar reactions to other drugs. For our patients, in the event of a polysorbate‐mediated reaction, slowing down the infusion could have allowed tolerance.

We suggest that clinicians measure levels of complement and interleukin‐6 (IL‐6) in cases of hypersensitivity reactions during infusion. If these levels are abnormal, patients would be expected to benefit from a slower infusion rate.^7^ The two major hypotheses behind these reactions are complement activation, which can respond to desensitization; or an interleukin/cytokine rash, which is similar to that observed with rituximab.^8^ This interleukin reaction is sensitive to the speed of infusion. Therefore, tolerance can be improved through slower administration of the initial infusion, which is the keystone to the desensitization protocol.

The Uppsala Monitoring Centre (UMC) has been mandated by the WHO to monitor drug safety since 1978. The UMC collates data from each national pharmacovigilance network of 172 member countries. Spontaneous notifications are collected into VigiBase, respecting the anonymity of both patients and notifiers. Collected data include sociodemographic characteristics (age, sex/gender, notifier's country) and details about the reported effect. VigiBase was queried on June 4, 2025, for all reports of hypersensitivity reactions with eptinezumab, defined by the *allergic reaction* high‐level group term from the Medical Dictionary for Regulatory Activities (MedDRA, version 28.0). MedDRA is a clinically validated international medical terminology dictionary used by regulatory authorities.

VigiBase documents 515 reports of potential immunoallergic reactions to eptinezumab, and disproportionality signals for “hypersensitivity” (185 cases vs. 70 expected; confidence interval CI_0.25_ = 1.2) and “infusion‐related reaction” (60 cases vs. 10 expected; CI_0.25_ = 2.1). Among the six French cases, all reactions occurred after the first administration, featuring early‐onset cutaneous or respiratory symptoms, consistent with trial‐reported infusion‐related reactions. This alignment stresses the reliability of pharmacovigilance data but also suggests underreporting or misclassification of immunological reactions, particularly when biomarkers are lacking. Current evidence remains insufficient to determine the precise mechanisms or recurrence risks upon re‐exposure. Clinically, infusion‐related reactions are similar to hypersensitivity reactions. An infusion‐related reaction would appear from the first exposure, and markers of hypersensitivity such as tryptase would be negative. In these cases, it would be recommended to first slow down the infusion and try administration under antihistamine to reduce nonspecific histamine release before proceeding with a desensitization. Notably, some patients in cases retrieved in VigiBase were concurrently exposed to NSAIDs, known cofactors of anaphylaxis.^9^ In patients with migraine, frequent NSAID use may lower the threshold of mast cell activation or increase the mucosal permeability and should be considered when interpreting these reactions. However, NSAIDS were not used in either of the two cases presented.

## CONCLUSION

These cases illustrate mild and moderate, but recurrent immediate hypersensitivity to eptinezumab, successfully managed via a first hospital‐based desensitization after the failure of premedication alone. The absence of severe clinical adverse effects and the therapeutic efficacy of eptinezumab justified this approach, which proved to be both feasible and effective.

The lack of immunological biomarker assessment highlights the need for standardized protocols (complement and IL‐6 assay) because the suspected pathophysiology directly affects the premedication strategy, monitoring, and design of desensitization.

## AUTHOR CONTRIBUTIONS

**Benoit Gerard:** Conceptualization; validation; writing – review and editing; investigation; writing – original draft. **Hubert Praudel:** Investigation; writing – original draft; validation; writing – review and editing. **Michel Lanteri‐Minet:** Investigation; validation; visualization; writing – review and editing. **Elise Van Obberghen:** Investigation; visualization; writing – review and editing; validation. **Fanny Rocher‐Moreau:** Investigation; methodology; validation; visualization; writing – review and editing; formal analysis. **Johanna Rousset:** Investigation; validation; methodology; visualization; writing – review and editing. **Ulysse Jacquier:** Validation; writing – review and editing. **Sylvie Leroy:** Validation; visualization; writing – review and editing; investigation. **Margot Delin:** Conceptualization; investigation; writing – original draft; methodology; validation; visualization; writing – review and editing; formal analysis; supervision.

## FUNDING INFORMATION

No funding was necessary.

## CONFLICT OF INTEREST STATEMENT

There was no conflict of interest for **Benoit Gerard**, **Hubert Praudel**, **Elise Van Obberghen**, **Fanny Rocher‐Moreau**, **Johanna Rousset**, **Ulysse Jacquier**, **Sylvie Leroy**, and **Margot Delin**. **Michel Lanteri Minet** has received personal fees for consultancy from: Abbvie/Allergan, Amgen, Eli Lilly, IPSEN, Lundbeck, Novartis, Orion Pharma, Perfood, Pfizer, Reckitt‐Benckiser, Teva, UPSA.
